# Identification and characterization of a novel homozygous splice site variant of *PATL2* causing female infertility due to oocyte germinal vesicle arrest

**DOI:** 10.3389/fgene.2022.967288

**Published:** 2022-08-22

**Authors:** Liwei Sun, Keya Tong, Weiwei Liu, Yin Tian, Sheng Yang, Danni Zhou, Dongyun Liu, Guoning Huang, Jingyu Li

**Affiliations:** ^1^ Chongqing Key Laboratory of Human Embryo Engineering, Center for Reproductive Medicine, Women and Children’s Hospital of Chongqing Medical University, Chongqing, China; ^2^ Chongqing Clinical Research Center for Reproductive Medicine, Chongqing Health Center for Women and Children, Chongqing, China

**Keywords:** female infertility, germinal vesicle arrest, PATL2, novel variant, aberrant splicing

## Abstract

**Background:** This study aims to describe clinical and diagnostic phenotype and identify pathogenic variants of a female with unknown causes of infertility.

**Methods:** Clinical assessment was performed for the phenotype diagnosis. Whole-exome sequencing (WES) and the followed cDNA-PCR sequencing were applied to identify the pathogenic variant and investigate the potentially aberrant mRNA splicing event. The pathogenicity of the variant was analysed using multiple *in silico* prediction tools, including the 3D protein remodelling. Quantitative RT-PCR (qRT-PCR) was performed to measure *PATL2* mRNA expression in the peripheral blood leukocytes of the proband and controls.

**Results:** The proband was diagnosed with the female infertility due to oocyte germinal vesicle (GV) arrest. A novel homozygous splice site variant of *PATL2* (NM_001145112.2, c.871-1G>A), inherited from her asymptomatic heterozygous parents, was detected by WES. Sequencing of cDNA amplification products demonstrated that this variant resulted in the exon 10 skipping and in-frame loss of 54 nucleotides in the *PATL2* transcript. Quantitative RT-PCR suggested that the mutant transcript escape the mRNA degradation.

**Conclusion:** We identified a novel pathogenic homozygous splice site of *PATL2* (c.871-1G>A) underlying the oocyte GV arrest phenotype and elucidated its molecular mechanism. This study expands the variant spectrum of *PATL2* and benefits our understanding of its genotype-phenotype correlations.

## Introduction

Infertility is a reproductive disorder that affects approximately 10–15% of couples worldwide (Maddirevula et al., 2020). A fundamental prerequisite for reproduction is oocyte maturation, which involves the stages from germinal vesicle (GV) to metaphase I (MI) and ultimately to metaphase II (MII) oocytes. Accordingly, oocyte maturation arrest (OMA) can occur at these different stages, including GV arrest, MI arrest, MII arrest, or mixed arrest, causing female infertility. OMA causes recurrent *in vitro* fertilization (IVF) or intracytoplasmic sperm injection (ICSI) failure; however, no therapeutic approaches are available due to our limited understanding of OMA causes (Beall et al., 2010). The phenotype of OMA was first described in 1990 (Rudak et al., 1990). The genetic causes underlying OMA were largely unknown until 2016, when variants in *TUBB8* (MIM: 616768) were reported to cause oocyte MI arrest (Feng et al., 2016a; Feng et al., 2016b; Chen et al., 2017a). Subsequently, *TRIP13* (MIM: 604507) was also identified as the causative gene for OMA (Zhang et al., 2020). It was also reported that variants in *PADI6* (MIM: 610363) cause female infertility characterized by early embryonic arrest (Xu et al., 2016). Variants in *WEE2* (MIM: 614084) are responsible for fertilization failure (Sang et al., 2018). Variants in *BTG4* (MIM: 605673) can cause zygotic cleavage failure and female infertility (Zheng et al., 2020).

Moreover, variants in *PATL2* (MIM: 614661; NM_001145112.2) can also cause female infertility. *PATL2* is an orthologue of *S. cerevisiae Pat1*. *PATL2* is located on 15q21.1 and encodes the protein PAT1 homologue 2, which is a mRNA-binding protein specifically expressed in immature oocytes that inhibits the post-transcriptional process in cells. As the oocyte matures, the expression of *PATL2* gradually disappears (Chen et al., 2017b). To date, 26 *PATL2* (NM_001145112.2) variants, including 21 nonsense variants and missense variants, 2 splicing variants, and 3 small deletions have been identified. However, pathogenic variants in *PATL2* were identified to result in GV arrest initially (Chen et al., 2017b; Maddirevula et al., 2017; Christou-Kent et al., 2018; Huang et al., 2018; Liu et al., 2020), and variable phenotypes were subsequently reported, including GV arrest, MI arrest, fertilization failure, and early embryonic arrest, indicating its high phenotypic heterogeneity (Wu et al., 2019). Although cases involving *PATL2* variants are accumulating, their molecular and pathophysiological mechanisms remains largely unknown. Therefore, more cases need to be investigated to illustrate phenotype-genotype relations and understand the molecular characteristics of *PATL2* variants.

Here, a novel homozygous splice site variant in *PATL2* that causes GV arrest was identified, and we revealed that this variant can result in exon skipping via aberrant splicing. Our study expands the variant spectrum of *PATL2* and benefits our understanding of its genotype-phenotype correlations and molecular aetiology.

## Materials and methods

### Ethical approval

In our study, the patients of primary female infertility with unknown reasons and control individuals (women with normal fertility) were recruited. All cases and control individuals with normal fertility were obtained from the Women and Children’s Hospital of Chongqing Medical University. Clinical information and peripheral blood samples were collected from the family after obtaining individual written informed consent. Genetic testing was performed in accordance with the Helsinki Declaration and approved by the ethics committee of Women and Children’s Hospital of Chongqing Medical University.

### Whole-exome sequencing and pathogenicity analysis

Genomic DNA was extracted from peripheral blood samples using a QIAamp DNA Blood Midi Kit (Qiagen, Hilden, Germany) according to the standard protocol. To detect the genetic causes of female infertility with oocyte germinal vesicle arrest, genetic variants were screened by whole-exome sequencing (WES) and confirmed by Sanger sequencing. Briefly, genomic DNA was captured using the Agilent SureSelectXT Human All Exon kit (Agilent Technologies, CA, United States) and sequenced on the MGISEQ-2000 platform with a 100 X read depth. Paired-end reads were aligned to the GRCh37/hg19 reference sequence using Burrows Wheeler Aligner software (BMA: version 0.7.8-r455). Variant calling was performed using SAMtools software (version 1.0). After variant detection, Annotate Variation (ANNOVAR) was used for variant annotation. Pathogenicity analysis of variants was performed according to the American College of Medical Genetics and Genomics (ACMG) practice guidelines. Variants were filtered based on the following criteria: 1) occurring in coding regions and/or splice sites; 2) nonsynonymous; 3) a frequency of less than 0.1% [Single Nucleotide Polymorphism database (dbSNP), Exome Variant Server, Genome Aggregation Database (gnomAD)]; 4) segregation with the phenotype. Sanger sequencing was used to verify the variants we screened and analyse the segregation data in the family. Pathogenicity prediction results of computational software (Human Splicing Finder 3.1 software (HSF), PROVEAN, MutationTaster, and CADD) were used to evaluate the effects of the variants on splicing.

### RNA extraction and cDNA sequencing

Total RNA was isolated from the peripheral blood leukocytes of the proband and control individuals by TRIzol LS reagent (Invitrogen, CA, United States). RNA was reverse-transcribed using a TaKaRa PrimeScript reagent kit (TaKaRa, Dalian, China) according to the manufacturer’s protocol. To investigate the abnormal splicing of the variant of *PATL2*, we amplified *PATL2* cDNA using primers spanning exons 8 to 15 in the proband and a normal control individual (NC). Then, the obtained PCR products were analysed by gel electrophoresis on a 1% agarose gel, and the further Sanger sequencing was performed on the cDNA amplified product. The PCR products were sequenced using ABI BigDye3.1 (Applied Biosystems, Foster City, CA, USA) and analysed using an ABI 3730XL sequencer.

### Quantitative RT–PCR

Quantitative RT–PCR (qRT–PCR) was conducted in a CFX96 real-time PCR detection system (Bio–Rad, Hercules, CA) by a final volume of 20 μl using SYBR Premix Ex Taq (Takara, Dalian, China) according to the manufacturer’s protocol. Glyceraldehyde 3-phosphate dehydrogenase (*GAPDH*) was served as an endogenous control. The relative mRNA expression levels were calculated using the 2−ΔΔCt method. All reactions were run in triplicate at a minimum, and data are presented as the means ± SD. Information on amplification for *PATL2* genomic splicing variant validation, cDNA sequencing and qPCR primers are shown in Supplementary Table S1.

### Molecular modelling and structural analysis

The 3D modelled structures of the PATL2 protein for the wild-type and mutant types were prepared using homology modelling in SWISS-MODEL (https://swissmodel.expasy.org/). Structural analysis and attribution of the residue interaction networks to the protein function were analysed and visualized using the PyMOL software (https://pymol.org/2/).

## Results

### Clinical manifestations

An individual diagnosed with primary infertility was recruited. The proband (II-2) was 34 years old and suffered from primary infertility for 9 years. Infertility-related examination did not reveal any physical abnormalities. Then, 225 IU of recombinant human follitropin was administered, and gonadotropin (Gn) was administered for 9 days. In addition, nine follicles with a diameter of at least 14 mm and one follicle with a diameter of 13 mm were achieved after human chorionic gonadotrophin (HCG) trigger administration, and eight oocytes were obtained. However, seven of them were arrested at the GV stage, and one oocyte was degenerated (Table 1).

**TABLE 1:** Gonadotrophin stimulation and follicular responses for the IVF cycle in the proband.

IVF cycle	
Male age (years)	34
Female age (years)	33
Infertility years	9
Basal hormones	
FSH(IU/L)	8.85
LH(IU/L)	2.82
E_2_ (pmol/L)	15.93
Prog (nmol/l)	0.3
PRL (ng/mL)	17.13
Hormones assay on day of HCG administration	
LH(IU/L)	1.8
E_2_ (pmol/L)	1744
FSH(IU/L)	10.6
Periods of FSH stimulation (days)	9
No. of leading follicles (≥18 mm)	4
No. of follicles (≥14 mm)	9

### Identification of a novel *PATL2* splice site variant

**FIGURE 1:**
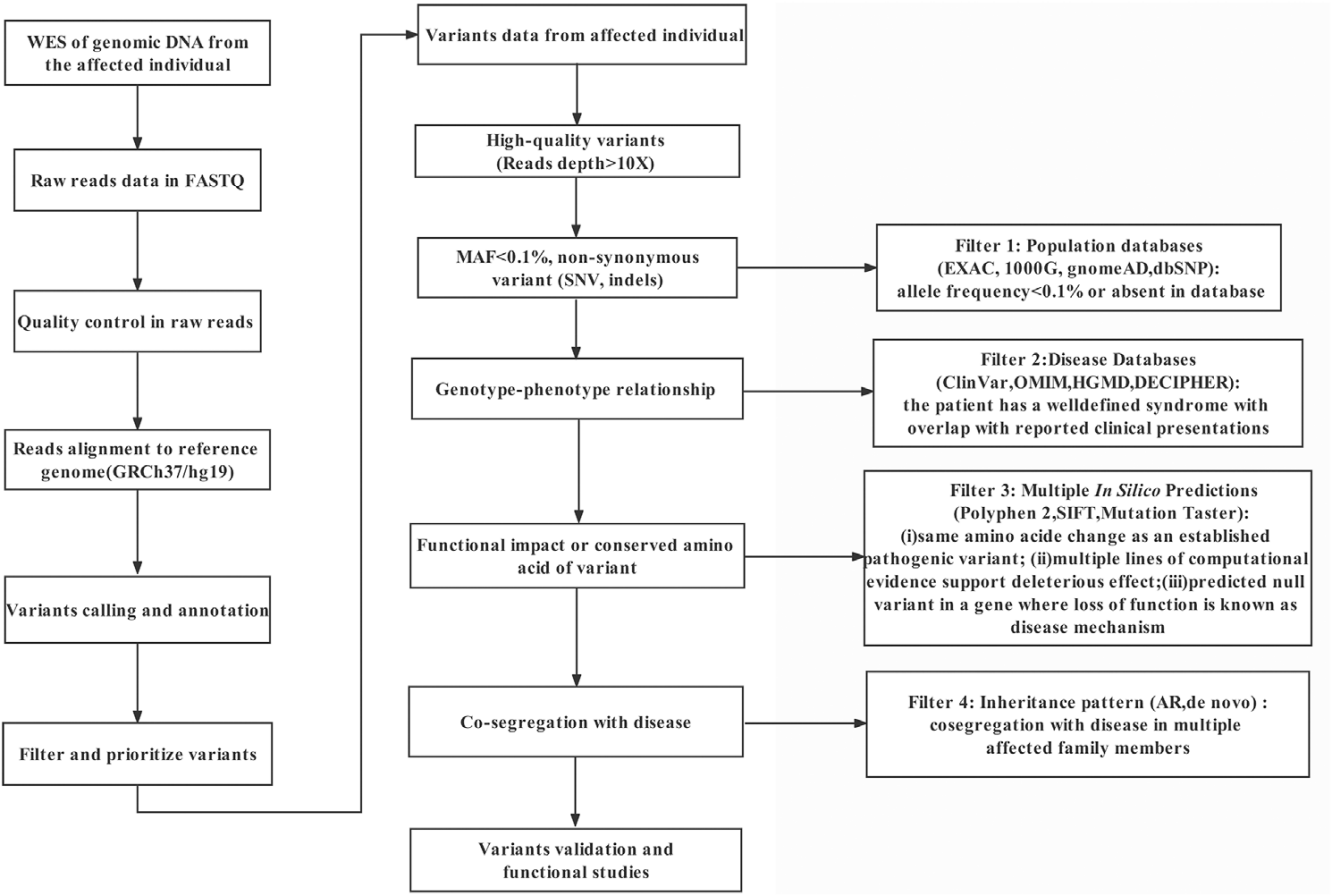
The analytical strategy workflow for variant filtration.

**FIGURE 2:**
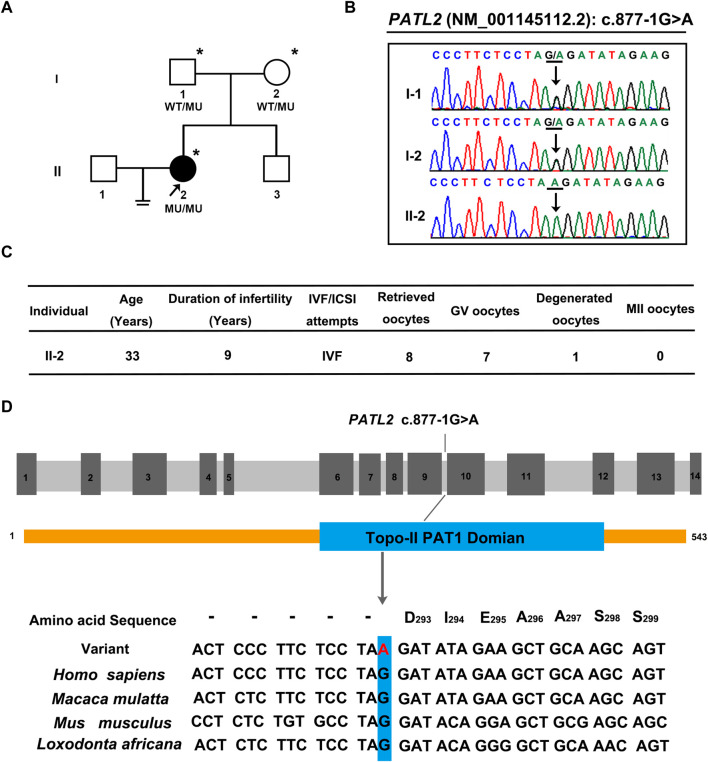
Pedigrees, clinical characteristics, and detection of variants in the female infertility family with oocyte germinal vesicle arrest. **(A)** Pedigrees of the affected family. The squares, circles, blackened, and open symbols indicate males, females, and affected and unaffected individuals, respectively. The arrows indicate the proband (II-2), and the asterisks denote the individuals who underwent genotyping. **(B)** Sequencing chromatograms of affected individuals who were homozygous for *PATL2* variant. Her father and mother were heterozygous carriers of the identified splicing variant. The black arrows indicate the sites of the variants. **(C)** clinical characteristic of affected individual and the retrieved oocytes in the IVF cycle. **(D)** The detected variant **(C)**871-1G>A in *PATL2* (MIM: 614661; GenBank: NM_001145112.2) in the affected family is evolutionarily conserved across different species. The site of *PATL2* variant is indicated in blue rectangle.

A novel homozygous splice site variant in the acceptor splice site of intron nine of *PATL2* (c.877-1G>A, NM_001145112.2) was detected in the proband, the WES analysis procedure was shown (Figure 1). Her father and mother were heterozygous carriers of the identified splice site variant (Figure 2A,B). The oocytes characteristics of IVF attempt of the proband was shown (Figure 2C). Locations of the splice site variant was denoted in the genomic structure and protein domains of *PATL2*. The *PATL2* splicing variant has a minor allele frequency of 6.76 × 10^–6^ within the global population and a minor allele frequency of 9.78 × 10^–5^ within the East Asian population in the gnomAD. The variant was considered deleterious by pathogenicity analysis using several in silico prediction tools, including HSF, MutationTaster, and CADD, which also predicted that the variant destroyed the acceptor site and most likely affected splicing. This variant detected in affected individual is also evolutionarily conserved across different species (Figure 2D).

### mRNA expression of mutant *PATL2*

**FIGURE 3:**
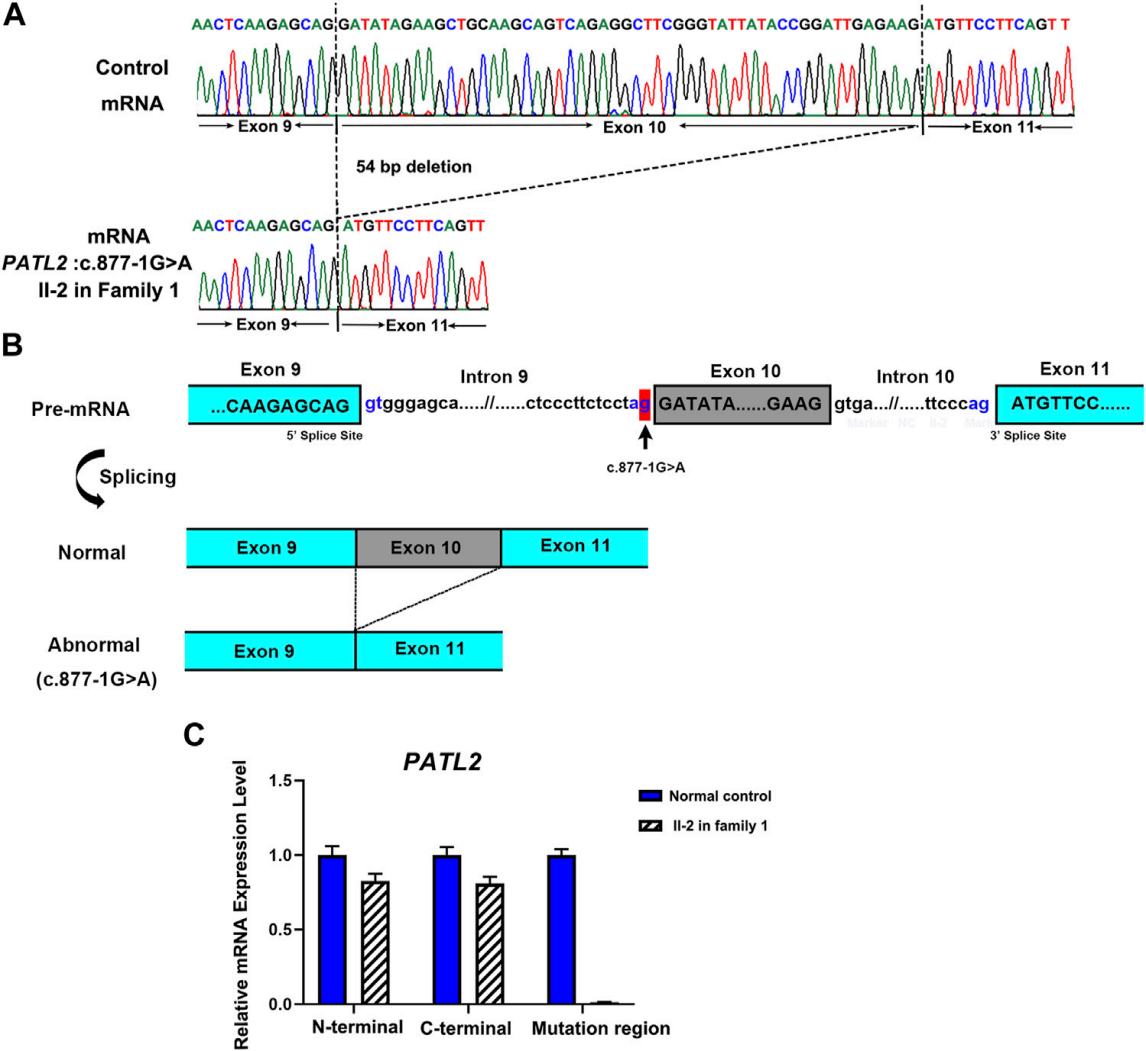
Functional analysis of the identified novel *PATL2* splice site variant. **(A)** Sanger sequencing traces of the RT–PCR products showed the wild-type (normal control, top) and *PATL2* exon 10 skipped transcripts (II-2 in the family, bottom). **(B)** Schematic representation of exon 9 to exon 11 of *PATL2* showing normal or abnormal splicing. The boxed regions denote exons, whereas the connecting lines indicate introns. **(C)** Quantitative RT–PCR (qRT–PCR) of the relative *PATL2* mRNA expression in peripheral blood lymphocytes from the affected individual (II-2) and the normal female controls (NC). For all qRT–PCR assays, glyceraldehyde 3-phosphate dehydrogenase (*GAPDH*) was used as an endogenous control. NC was set to 1.0, and data are presented as the mean ± SD (*n* ≥ 3).

To investigate the abnormal splicing result of *PATL2* mRNA, Sanger sequencing of the *PATL2* cDNA was performed, and the exon 10 skipping was observed (Figure 3A). The aberrant splicing of this novel splice site variant was also denoted (Figure 3B). Furthermore, three pairs of *PATL2* primers were designed for qPCR, including one pair of primer downstream at the exon 10 region, and two pairs of primers spanning the N-termination and C-termination which do not include the variant region, respectively. As a result, no detectable mRNA expression level was showed in the proband at the exon 10 region, and the splicing variant would not introduce premature translation stop codons or result in *PATL2* mRNA decay, which further confirmed the abnormal exon 10 skipping results (Figure 3C).

### Protein structure modelling

**FIGURE 4:**
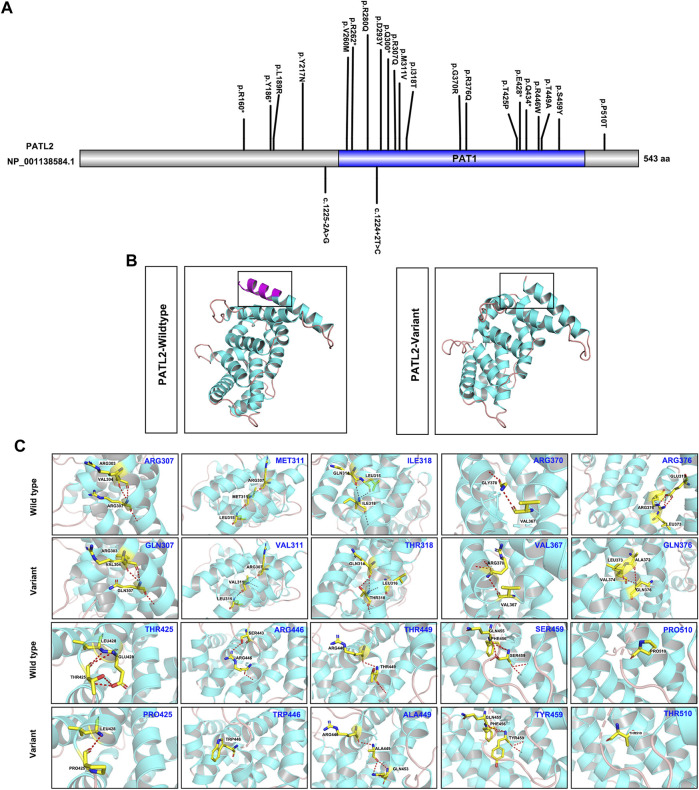
Schematic diagrams of the locations of reported *PATL2* variants and molecular modelling of wild-type and mutant PATL2 proteins. **(A)** The variants marked are the *PATL2* variants reported to date, and the novel variant identified in the present study is marked and indicated in red. **(B)** Three-dimensional schematic of the structure of normal PATL2 protein (on the left) and the mutant PATL2 (on the right). A 18-amino-acid in-frame deletion p. (Asp293_Lys310) in the PAT1 domain of the PATL2 protein is the putative result of the homozygous splicing variant **(C)**871-1G>A. The alteration in the three-dimensional positioning of the splicing variant causes steric hindrance to the formation of α-helix structures in the PATL2 protein, which are highlighted in pink. **(C)** Three-dimensional schematic of the structure of the previous reported missense variants of *PATL2*.

To explore the phenotype-genotype relationship, we located the reported *PATL2* variants in the PATL2 protein and summarized genotype-phenotype correlations based on previous reports (Figure 4A). In addition to the result that the homozygous splicing variant c.871-1G>A causes the exon 10 skipping at the mRNA level, protein structure modelling also revealed that the alteration in the three-dimensional positioning of the splicing variant causes steric hindrance to the formation of α-helix structures in the PATL2 protein, which are highlighted in pink (Figure 4B). The *PATL2* variants reported and the phenotype including the ratio of GV arrest were characterized (Table 2). Effect of reported *PATL2* variants on the protein structure were also modelled (Figure 4C).

**TABLE 2:** Characteristics of patients with *PATL2* variants and results of IVF cycle.

Variant	Amino acid change	Inheritance	Age (Years)	Main phenotype	Duration of infertility (Years)	IVF and ICSI cycles	Total oocytes retrieved	GV oocytes	MI oocytes	PB1 oocytes	References
c.478C>T	p. R160*	Homozygous	23	70/79 GV arrest	7	7	79	70	—	—	Maddirevula et al. (2017)
				7/79 degenerated							
			35	4/5 GV arrest	—	3	5	4	0	0	Christou-Kent et al. (2018)
				1/5 degenerated							
			28	24/39 GV arrest	—	2	39	24	0	0	
				25/39 degenerated							
			24	11/16 GV arrest	—	1	16	11	0	0	
				5/16 degenerated							
			34	11/16 GV arrest	—	1	10	8	0	0	
				5/16 degenerated							
			41	5/9 GV arrest	—	2	9	5	1	0	
				3/9 degenerated							
			36	2/21 GV arrest, 10/21 cytoplasmic vacuoles in MI oocyte, 9/21 degenerated	—	2	21	2	10	0	
c.558T>A	p.Y186*	Compound heterozygous	32	2/3 GV arrest	9	1	3	2	0	0	Chen et al. (2017b)
c.223-14_223-2del13	Not yet available										
c.566T>G	p.L189R	Compound heterozygous	30	13/75 GV arrest	6	3	75	13	14	18	Chen et al., 2017b)
c.649T>A	p.Y217N										
c.649T>A	p.Y217N	Compound heterozygous	32	4/6 MI arrest	4	1	6	0	4	2	Cao et al. (2021)
c.920G>A	p.R307Q										
c.1108G>A	p.G370R	Homozygous	25	20/20 GV arrest	6	5	20	20	—	—	Maddirevula et al. (2017)
c.784C>T	p.R262*	Homozygous	31	4/5 GV arrest	8	1	5	4	1	0	Chen et al. (2017b)
c.953T>C	p.I318T	Compound heterozygous	31	4/64 GV arrest, 7/64 MI arrest, 49/64 abnormal large PB1 oocytes or abnormal cleavage or early embryonic stage arrest	11	8	64	4	7	49	Chen et al., 2017b)
c.839G>A	p.R280Q										
c.649T>A	p.Y217N	Compound heterozygous	30	13/75 GV arrest, 14/75 MI arrest, 18/75 abnormal large PB1 oocytes/abnormal cleavage/early embryonic stage arrest	6	3	75	13	14	18	Chen et al. (2017b)
c.566T>G	p.L189R										
c.223-14_223-2del13	Not yet available	Compound heterozygous	33	18/47 GV arrest, 21/47 abnormal cleavage/early embryonic stage arrest	3	3	47	18	2	21	Chen et al. (2017b)
c.1224+2T>C	—										
c.1127G>A	p. R376Q	Homozygous	32	All in GV arrest	6	3	32	32	NA	NA	Huang et al. (2018)
c.223-14_223-2del13	Not yet available	Compound heterozygous	35	All in GV arrest	7	2	17	17	NA	NA	Huang et al. (2018)
c.1225-2A>G											
c.1282G>T	p.E428*	Compound heterozygous	27	All in GV arrest	4	3	21	21	NA	NA	Huang et al. (2018)
c.1300C>T	p.Q434*										
c.1282G>T	p.E428*	Compound heterozygous	33	All in GV arrest	4	2	40	40	NA	NA	Huang et al. (2018)
c.865delA	p. T289Lfs*6										
c.898C>T	p.Q300*	Compound heterozygous	28	6/24 in GV arrest	4	2	24	6	0	0	Wu et al. (2019)
c.1273A>C	p. T425P										
c.898C>T	p.Q300*	Heterozygous	25	17/33 in GV arrest	3	3	33	17	0	0	Cao et al. (2021)
				16/33 degenerated							
c.877G>T	p. D293Y	Compound heterozygous	32	6/42 in MI arrest	8	4	42	0	0	6	Wu et al. (2019)
c.223-14_223-2del13	Not yet available										
c.778G>A	p. V260M	Compound heterozygous	27	All in GV arrest	4	1	11	11	0	0	Wu et al. (2019)
					10	3	19	0	2	17	Cao et al. (2021)
c.223-14_223-2del13	Not yet available			17/19 in MI arrest							
c.1528C>A	p. P510T	Homozygous	39	62/71 in GV arrest	15	2	71	62	2	—	Liu et al. (2020)
c.1345A>G	p. T449A	Heterozygous	32	No GV arrest, 4/6 MI arrest	4	2	6	0	4	2	Cao et al. (2021)
c.1376C>A	p. S459Y	Heterozygous	28	4/5 MI oocytes	3	1	5	0	4	1	Cao et al. (2021)
				1/5 matured Pb1 oocyte							
c.1376C>A	p. S459Y	Homozygous	37	8/18 in GV arrest	13	3	18	8	8	0	Liu et al. (2020)
c.1336C>T	p. Arg446Trp	Heterozygous	31	All in GV arrest	7	1	12	12	0	0	Cao et al. (2021)
c.920G>A	p. Arg307Gln	Compound heterozygous	32	4/6 MI arrest	4	1	6	0	4	2	Cao et al. (2021)
c.649T>A	p. Tyr217Asn										
c.1376C>A	p. Ser459Tyr	Compound heterozygous	28	17/30 in GV arrest	7	2	30	17	5	3	Cao et al. (2021)
c.931A>G	p. Met311Val										

GV, germinal vesicle; MI, metaphase I; PB1, first polar body.

## Discussion

Several clinical reports on pathogenic variants of *PATL2* in OMA patients have been published. In our study, the majority of the oocytes retrieved from the proband were arrested at the GV stage, which was consistent with the typical phenotype from previously reported *PATL2* variants (Chen et al., 2017b; Maddirevula et al., 2017; Christou-Kent et al., 2018; Huang et al., 2018; Wu et al., 2019; Liu et al., 2020; Cao et al., 2021). To date, only two splicing variants have been reported, including c.1225-2A>G and c.1224 + 2T>C. The splicing variant c.1225-2A>G was predicted to abolish the canonical splice acceptor site of exon 13, and c.1224 + 2T>C was predicted to contribute to PATL2 protein truncation (Chen et al., 2017b; Huang et al., 2018). The two reported splicing variants were all compound heterozygous variants. To the best of our knowledge, we report a novel homozygous splicing variant for the first time. To understand the molecular pathogenesis of this novel *PATL2* splicing variant of c.877-1G>A, we demonstrated that this variant may cause exon 10 of *PATL2* to be skipped at the mRNA level and lead to a 18-amino-acid in-frame deletion p. (Asp293_Lys310) in the PAT1 domain of the PATL2 protein, which may result in loss of the RNA-binding ability. Additionally, we also confirmed that this variant did not introduce a premature termination codon (PTC) or trigger nonsense-mediated decay (NMD) of the truncated mRNA. Immunostaining analysis using oocytes from the affected individual may help to evaluate the possible effects of the novel variant on the PATL2 protein level. However, it is unfortunate that no oocyte was available for such analyses in the present study.

*PATL2* was initially regarded as a mRNA-binding protein (mRNP) associated with other mRNPs, such as Xp54, xRAP55, and CPEB (Radford et al., 2008; Nakamura et al., 2010). Previous studies have reported that variants in *PATL2* lead to female infertility with oocyte maturation arrest; however, the mechanisms by which *PATL2* variants affect meiotic maturation remain unclear and controversial. It was confirmed that affected individuals with *PATL2* variants harbour loss-of-function variants, and the oocytes of some individuals showed decreased on PATL2 protein levels. These findings imply that the variants cause accelerated protein degradation instead of activation (Chen et al., 2017b). Another study recently revealed that some *PATL2* variants lead to abnormally increased *PATL2*-bound mRNAs in Mos, an upstream activator of mitogen-activated protein kinase (MAPK), to regulate its translational activity and subsequently impair the MAPK signalling pathway and oocyte meiosis (Cao et al., 2021). These results highlighted the role of *PATL2* in translational regulation of Mos and its association with the MAPK signalling pathway during oocyte meiotic maturation. Subsequently, we analysed the clinical characteristics of the cases in the present study and published cases associated with *PATL2* variants. Therefore, combined with our finding that the novel homozygous variants c.877-1G>A in *PATL2* reduce *PATL2* mRNA expression levels, we inferred that the diverse pathogenic mechanisms of *PATL2* over expression, deletion or bidirectional effects of *PATL2* may be involved in the oocyte maturation process. These hypotheses are worthy of further investigation to elucidate the genetic mechanisms involved.

In conclusion, we have identified a novel homozygous splice site variant of *PATL2* in a female individual affected by oocyte maturation arrest with GV arrest. We also provided evidence for the effects of the splice site variant on *PATL2* mRNA expression levels to molecularly characterize its role in the oocyte GV arrest phenotype. Our study expands the variant spectrum of oocyte maturation arrest patients with *PATL2* variants and benefits our understanding of genotype-phenotype correlations of *PATL2*.

## Data Availability

The data presented in the study are deposited in the Genome Sequence Archive (GSA) repository, accession number subHRA003831.

## References

[B1] Beall S. Brenner C. Segars J. (2010). Oocyte maturation failure: A syndrome of bad eggs. Fertil. Steril. 94 (7), 2507–2513. 10.1016/j.fertnstert.2010.02.037 20378111 PMC2946974

[B2] Cao Q. Zhao C. Wang C. Cai L. Xia M. Zhang X. (2021). The recurrent mutation in PATL2 inhibits its degradation thus causing female infertility characterized by oocyte maturation defect through regulation of the mos-MAPK pathway. Front. Cell Dev. Biol. 9, 628649. 10.3389/fcell.2021.628649 33614659 PMC7890943

[B3] Chen B. Li B. Li D. Yan Z. Mao X. Xu Y. (2017a). Novel mutations and structural deletions in TUBB8: Expanding mutational and phenotypic spectrum of patients with arrest in oocyte maturation, fertilization or early embryonic development. Hum. Reprod. 32 (2), 457–464. 10.1093/humrep/dew322 27989988

[B4] Chen B. Zhang Z. Sun X. Kuang Y. Mao X. Wang X. (2017b). Biallelic mutations in PATL2 cause female infertility characterized by oocyte maturation arrest. Am. J. Hum. Genet. 101 (4), 609–615. 10.1016/j.ajhg.2017.08.018 28965849 PMC5630194

[B5] Christou-Kent M. Kherraf Z. E. Amiri-Yekta A. Le Blevec E. Karaouzene T. Conne B. (2018). PATL2 is a key actor of oocyte maturation whose invalidation causes infertility in women and mice. EMBO Mol. Med. 10 (5), e8515. 10.15252/emmm.201708515 29661911 PMC5938616

[B6] Feng R. Sang Q. Kuang Y. Sun X. Yan Z. Zhang S. (2016a). Mutations in TUBB8 and human oocyte meiotic arrest. N. Engl. J. Med. 374 (3), 223–232. 10.1056/NEJMoa1510791 26789871 PMC4767273

[B7] Feng R. Yan Z. Li B. Yu M. Sang Q. Tian G. (2016b). Mutations in TUBB8 cause a multiplicity of phenotypes in human oocytes and early embryos. J. Med. Genet. 53 (10), 662–671. 10.1136/jmedgenet-2016-103891 27273344 PMC5035199

[B8] Huang L. Tong X. Wang F. Luo L. Jin R. Fu Y. (2018). Novel mutations in PATL2 cause female infertility with oocyte germinal vesicle arrest. Hum. Reprod. 33 (6), 1183–1190. 10.1093/humrep/dey100 29697801

[B9] Liu Z. Zhu L. Wang J. Luo G. Xi Q. Zhou X. (2020). Novel homozygous mutations in PATL2 lead to female infertility with oocyte maturation arrest. J. Assist. Reprod. Genet. 37 (4), 841–847. 10.1007/s10815-020-01698-6 32048119 PMC7183019

[B10] Maddirevula S. Awartani K. Coskun S. AlNaim L. F. Ibrahim N. Abdulwahab F. (2020). A genomics approach to females with infertility and recurrent pregnancy loss. Hum. Genet. 139 (5), 605–613. 10.1007/s00439-020-02143-5 32172300

[B11] Maddirevula S. Coskun S. Alhassan S. Elnour A. Alsaif H. S. Ibrahim N. (2017). Female infertility caused by mutations in the oocyte-specific translational repressor PATL2. Am. J. Hum. Genet. 101 (4), 603–608. 10.1016/j.ajhg.2017.08.009 28965844 PMC5630161

[B12] Nakamura Y. Tanaka K. J. Miyauchi M. Huang L. Tsujimoto M. Matsumoto K. (2010). Translational repression by the oocyte-specific protein P100 in Xenopus. Dev. Biol. 344 (1), 272–283. 10.1016/j.ydbio.2010.05.006 20471969

[B13] Radford H. E. Meijer H. A. de Moor C. H. (2008). Translational control by cytoplasmic polyadenylation in Xenopus oocytes. Biochim. Biophys. Acta 1779 (4), 217–229. 10.1016/j.bbagrm.2008.02.002 18316045 PMC2323027

[B14] Rudak E. Dor J. KiMchiM. Goldman B. Levran D. MaShiach S. (1990). Anomalies of human oocytes from infertile women undergoing treatment by *in vitro* fertilization. Fertil. Steril. 54 (2), 292–296. 10.1016/s0015-0282(16)53706-6 2379628

[B15] Sang Q. Li B. Kuang Y. Wang X. Zhang Z. Chen B. (2018). Homozygous mutations in WEE2 cause fertilization failure and female infertility. Am. J. Hum. Genet. 102 (4), 649–657. 10.1016/j.ajhg.2018.02.015 29606300 PMC5985286

[B16] Wu L. Chen H. Li D. Song D. Chen B. Yan Z. (2019). Novel mutations in PATL2: Expanding the mutational spectrum and corresponding phenotypic variability associated with female infertility. J. Hum. Genet. 64 (5), 379–385. 10.1038/s10038-019-0568-6 30765866

[B17] Xu Y. Shi Y. Fu J. Yu M. Feng R. Sang Q. (2016). Mutations in PADI6 cause female infertility characterized by early embryonic arrest. Am. J. Hum. Genet. 99 (3), 744–752. 10.1016/j.ajhg.2016.06.024 27545678 PMC5010645

[B18] Zhang Z. Li B. Fu J. Li R. Diao F. Li C. (2020). Bi-Allelic missense pathogenic variants in TRIP13 cause female infertility characterized by oocyte maturation arrest. Am. J. Hum. Genet. 107 (1), 15–23. 10.1016/j.ajhg.2020.05.001 32473092 PMC7332649

[B19] Zheng W. Zhou Z. Sha Q. Niu X. Sun X. Shi J. (2020). Homozygous mutations in BTG4 cause zygotic cleavage failure and female infertility. Am. J. Hum. Genet. 107 (1), 24–33. 10.1016/j.ajhg.2020.05.010 32502391 PMC7332666

