# Supplementary material for: Identification and characterization of a novel homozygous splice site variant of PATL2 causing female infertility due to oocyte germinal vesicle arrest
Source: Front Genet. 2022 Aug 22;13:967288. doi: 10.3389/fgene.2022.967288 (PMC9441802; doi:10.3389/fgene.2022.967288)
Supplement: Supplementary file 1 [file Table1.DOCX]

| **Primers** | **Primers Sequence** | **Fragment size** |
| --- | --- | --- |
| *PATL2*- Primer 1  For variant validation | Forward: AAGAGCAGGTGGGAGCACTA | 369bp |
|  | Reverse: GGGAATTTGAGGGTGGAAAA |  |
| *PATL2*-Primer 2  For cDNA sequencing | Forward: GAGAAGAAGCAGGCAGAC | 813bp |
|  | Reverse: GGCAATCAGAACCACCAT |  |
| *PATL2*- Primer 3  For qPCR (N termination) | Forward: AGGAGGAGGAAGAGGAGGAG | 178bp |
|  | Reverse: GCAAGTGACATTCCCAGCAT |  |
| *PATL2*- Primer 4  For qPCR (C termination) | Forward: ACTTGACCCTCCACGAACTC | 188bp |
|  | Reverse: ATGGTCACTGTTGGGTTCCT |  |
| *PATL2*- Primer 5  For qPCR (Variant region) | Forward: CGCCTGGATGACTACTATTACC | 280bp |
|  | Reverse: ATACCCGAAGCCTCTGACTG |  |
| *GAPDH*  For qPCR | Forward: TGCACCACCAACTGCTTAG | 176bp |
|  | Reverse: CAGGCAGGGATGATGTTC |  |

**Table S1. Amplification for *PATL2* splicing genomic variant validation, cDNA sequencing and qPCR primers.**
